# Adaptive Beamforming, Cell-Free Resource Allocation and NOMA in Large-Scale Wireless Networks

**DOI:** 10.3390/s24237548

**Published:** 2024-11-26

**Authors:** Panagiotis Gkonis, Spyros Lavdas, George Vardoulias, Panagiotis Trakadas, Lambros Sarakis, Konstantinos Papadopoulos

**Affiliations:** 1 Department of Digital Industry Technologies, National and Kapodistrian University of Athens, Dirfies Messapies, 34400 Athens, Greece; lsarakis@uoa.gr (L.S.); konspap@uoa.gr (K.P.); 2 Department of Information Technology, American College of Greece, Ag. Paraskevi, 15342 Athens, Greece; slavdas@acg.edu; 3 Hellenic Naval Academy, 18539 Piraeus, Greece; gvardoulias@hna.gr; 4 Department of Port Management and Shipping, National and Kapodistrian University of Athens, Dirfies Messapies, 34400 Athens, Greece; ptrakadas@uoa.gr

**Keywords:** 5G, non-orthogonal multiple access, massive MIMO, millimeter wave transmission, system-level simulations

## Abstract

The goal of the study presented in this work is to evaluate the performance of a proposed adaptive beamforming approach when combined with non-orthogonal multiple access (NOMA) in cell-free massive multiple input multiple output (CF m-MIMO) orientations. In this context, cooperative beamforming is employed taking into consideration the geographically adjacent access points (APs) of a virtual cell, aiming to minimize co-channel interference (CCI) among mobile stations (MSs) participating in NOMA transmission. Performance is evaluated statistically via extensive Monte Carlo (MC) simulations in a two-tier wireless orientation. As the results indicate, for high data rate services, various key performance indicators (KPIs) can be improved compared to orthogonal multiple access, such as the minimum number of users in the topology as well as the available PRBs for downlink transmission. Although in NOMA transmission more directional beamforming configurations are required to compensate for the increased CCI levels, the increase in the number of hardware elements is reduced compared to the corresponding gain in the considered KPIs.

## 1. Introduction

The full deployment of fifth generation (5G) networks is expected to leverage the delivery of advanced services and applications to end users, such as enhanced mobile broadband (eMBB), ultra-reliable low latency communications (URLLC) and massive machine-type communications (mMTC) [1]. The 5G era is based on various novel technologies both in the physical and network layer, such as massive multiple input multiple output (m-MIMO) configurations [2], millimeter wave (mmWave) transmission [3], non-orthogonal multiple access (NOMA) [4], and network function virtualization (NFV) [5]. When m-MIMO is combined with mmWave transmission, multiple antenna arrays can be deployed in various access points (APs) of the wireless orientation. Hence, highly directional beams can be formulated, improving spectral and energy efficiency (SE, EE).

However, the rapid growth in the number of interconnected devices on the internet (internet of things—IoT), along with the ever-increasing user requirements for high data rates and URLLC in densely deployed networks, necessitates the deployment of new transmission protocols and architectural approaches to fulfill these demands. In this context, the goal is to improve various key performance indicators (KPIs), with minimum hardware and computational burden. The optimization of a multitude of KPIs is extremely important for the design and implementation of future broadband networks (sixth generation—6G) where the concept of ultra dense networks (UDNs) has been proposed, among others, as a candidate architectural approach that can leverage eMBB and URLLC [6].

In NOMA transmission, the same resource block (e.g., physical resource block—PRB) is utilized concurrently by individual mobile stations (MSs). In this context, a challenging issue is the selection of groups of MSs to minimize co-channel interference (CCI). In cooperative NOMA, an MS with improved channel conditions may act as an intermediate relay node that forwards the requested information to another MS at the edge of the network, in the case where the direct link from the AP would significantly increase the overall transmission power. The optimum selection of NOMA MSs (referred to as the NOMA group—NG—throughout the rest of this paper) can improve various KPIs, since more PRBs are available for uplink/downlink transmission without additional spectrum requirements.

### 1.1. Related Works

Over the past few years, various scientific works have dealt with the deployment of NOMA transmission in m-MIMO configurations. In [7], the non-convex optimization problem of sum-rate and energy efficiency maximization was formulated for m-MIMO NOMA systems with hybrid precoding. In this context, the MS grouping metric takes into account two features, namely the MS channel vector and the distance between MSs. Due to the non-convex nature of the problem, the linked variables, such as power allocation and power splitting ratio assignments, were decoupled. According to the presented results, the proposed approach can significantly improve SE and EE compared to other state-of-the-art approaches. However, per user optimum channel assignment has not been considered. In [8], the problem of joint task assignment, power allocation and node grouping is considered for NOMA–mmWave multi-access edge computing (MEC) environments. To this end, a device that is located in close proximity to the considered MS may also perform local computations and upload the results to the closest MEC server. This device is referred to as the helper. Afterwards, an optimization problem is formulated which is solved in two steps: In the first step, a low complexity search algorithm is employed to select the helper and the MEC node. The second step includes energy consumption minimization. The non-convex problem is later solved via sub-optimal approaches. According to the presented results, the proposed cooperative NOMA approach can significantly improve energy consumption compared to other existing works.

In [9], a performance evaluation of NOMA transmission in multicellular MIMO orientations takes place via a developed hybrid system–link-level simulator. To this end, MS grouping takes into consideration the maximization of the signal to interference plus noise ratio (SINR) as well as the signal to jamming ratio (SJR). According to the presented results, if up to 20% of the available PRBs are shared for NOMA transmission, the need for successive interference cancelation (SIC) at the receiver is practically eliminated. However, unavoidably, the calculation of SINR and SJR on large-scale networks can be a time-consuming processe that requires increased signaling burden. In [10], intelligent reflecting surface (IRS)–NOMA-assisted cell-free (CF) m-MIMO systems are considered. The overall optimization problem can be quite complex, since it involves the joint optimization of power allocation, phase shifts in IRSs, and MS pairing. Therefore, deep reinforcement learning approaches have been presented. The results indicate that the incorporation of NOMA into the IRS-assisted CF m-MIMO system and the use of the proposed Deep Deterministic Policy Gradient-based optimization algorithm can significantly improve downlink transmission rate. In the same context (i.e., IRS-NOMA), in [11], an adaptive MS pairing algorithm is considered, where two MSs with different channel characteristics are grouped in the same NG. In this context, multiple IRSs help to improve the signal quality received. A deep learning (DL) framework is proposed to find the optimum solution in the joint optimization problem of precoding the matrix formulation, phase shifts in IRSs, and the involved NOMA MSs. It is shown that the proposed DL approach can significantly reduce overall execution times when compared to classical optimization methods.

In [12], the performance of a spatial modulation system using NOMA transmission is evaluated over multiple subchannels. In this context, a joint subchannel and power allocation problem for weighted sum rate maximization is formulated, which is decomposed into three subproblems, namely the decoding order design, the subchannel assignment, and the power allocation. In [13], a tier-based NOMA clustering approach is presented. To this end, three different cases are evaluated: groups of two users, groups of three users and OMA. The final selection is based on the distance of the candidate users from their serving BS. According to the presented results, the achievable sum rate can be improved in the case of NOMA. However, the results are limited in a single type of service per user. In [14], a DRL approach is considered for optimal user clustering in internet of remote things-oriented satellite terrestrial relay networks. To this end, the goal is to maximize the overall sum rate capacity. As the authors point out, the computational complexity can increase exponentially with network size. In [15], the authors have focused on the detrimental effect of CCI from nearby NOMA transmissions towards a relay-aided NOMA network. In greater detail, randomly located CCI terminals near the relay-aided network use NOMA to communicate, thus degrading the uplink communication performance. In this setting, optimization problems are formulated for the transmit power, power allocation and relay location, providing improved performance by reducing the impact of CCI.

Finally, in [16] NOMA is employed in unmanned aerial vehicles (UAVs), where the UAV acts as a relay to assist the ground MSs. An optimization problem is formulated to determine the optimum beamforming configuration and position of the UAV. This problem was decomposed to two subproblems that were solved via successive convex approximation techniques. The results indicate that the sum rate can be improved with respect to the conventional NOMA case, where MSs are served by fixed APs.

### 1.2. Contributions

The key outcomes of the presented works are summarized in Table 1. In all the aforementioned studies, either limited network deployments have been considered (i.e., limited number of users or APs) or the combination of NOMA with m-MIMO configurations has not been thoroughly studied. Furthermore, the majority of the related studies have not taken realistic antenna radiation patterns into account. In this work, we propose and evaluate a low-complexity NG selection algorithm that can enhance various performance metrics, such as the minimum number of supported MSs in the network, and available PRBs for downlink transmission, while minimizing hardware requirements and computational overhead. The main contributions of our work are summarized as follows:

- NOMA transmission is considered in multi-AP m-MIMO orientations (two tiers of cells around the central cell). In this case, the goal is to effectively manage CCI via MIMO-enabled transceiver techniques.
- CF resource allocation (RA) is also considered, where an MS can now be served by multiple APs according to channel conditions and CCI levels, thus leveraging effective resource management.
- Since interference management in large-scale CF m-MIMO orientations can be quite a challenging task (RA involves multiple APs), a cooperative beamforming approach is presented as well. This approach jointly considers the APs that participate in RA, trying to achieve a dual goal: on the one hand to effectively support NOMA transmission by minimizing interference from adjacent APs and on the other hand to minimize the number of required hardware elements for downlink transmission.
- Unlike other works in the literature, MSs in an NG are assigned with the PRBs that maximize their signal strength and CCI is minimized via cooperative beamforming.
- mmWave transmission is also considered, according to the latest 3GPP guidelines and specifications [17]. In particular, the deployed antenna radiation diagrams correspond to an 28 GHz carrier frequency.

This work is a continuation of the work initially presented in [18], where NOMA transmission is now also considered in the CF m-MIMO multi-AP orientation. The rest of this paper is organized as follows: In Section 2, the 5G mmWave CF m-MIMO orientation is presented, while in Section 3 the antenna design aspects per AP are highlighted. In Section 4, the proposed resource allocation approach based on NOMA transmission and cooperative beamforming is analyzed. The results are presented in Section 5, where various KPIs have been considered. Finally, concluding remarks are provided in Section 6.

The following notation is used throughout the paper. An italic variable *a* denotes a scalar, whereas boldface lowercase and uppercase variables **a** and **A** denote vectors and matrices, respectively. A calligraphic variable A denotes a set of $\left|A\right|$ elements. **A**^T^ and **A**^H^ denote the transpose and conjugate transpose of **A**, respectively, while ${\left\|x\right\|}_{F}$ stands for the Frobenius norm of **x**. Finally, **A**(*i*,*j*) is the (*i*,*j*) entry of the two-dimensional matrix **A**.

## 2. Fifth-Generation Massive MIMO Millimeter Wave Cell-Free Orientations

**Figure 1:**
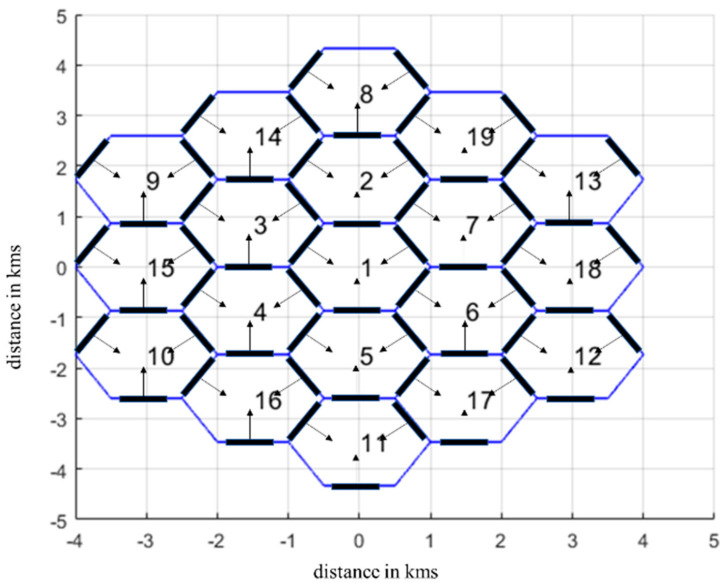
m-MIMO orientation with multiple access points and virtual cells.

We consider the downlink of a multi-AP wireless orientation, with *N* APs uniformly distributed (the pointing direction of each AP’s coverage area is illustrated with the help of arrows). To this end, virtual cells (VCs) are formulated, as shown in Figure 1. Each AP is equipped with an m-MIMO configuration, as will be described in detail in the following section. The transmitted signal can be expressed as

(1) $$x_{k}(t)=\sum_{s\in U_{k}}\sqrt{p_{k,T(k,s),s}}t_{k,T(k,s),s}X_{k,s}e^{j2\pi f_{s}t},0<t<T_{s},$$

where $U_{k}$ is the set of PRBs assigned to the *k*th MS. It should be noted at this point that in the CF operational mode, an MS can be served by multiple APs according to channel conditions. The mapping of APs and PRBs for all active MSs is stored in the two-dimensional matrix T. Therefore, T(*k*,*s*) denotes the AP that serves the *k*th MS with respect to the *s*th PRB. Assuming *M_t_* transmitting antennas for the AP of interest, then t*_k_*_,T(*k*,*s*),*s*_ is the *M_t_* × 1 transmission vector (diversity combining mode has been assumed), *p_k_*_,T(*k*,*s*),*s*_ is the allocated power to the *s*th PRB of the *k*th MS and X*_k_*_,*s*_ is the transmission symbol selected from a predefined constellation (i.e., QPSK, 16QAM, 64QAM). Finally, *T_s_* is the symbol period, and *f_s_* is the corresponding frequency of the *s*th PRB.

The received *M_r_* × 1 signal (*M_r_* is the number of receiving antennas) per MS’s PRB is a superposition of all co-channel signals from all other APs (intercell co-channel interference, Inter-CCI) as well as from the same AP in the case of NOMA transmission (intracell co-channel interference, Intra-CCI):

(2) $$\begin{array}{l} Y_{k,s}=\left(\sqrt{\frac{p_{k,T(k,s),s}}{TL_{k,T(k,s)}}}\right)r_{k,T(k,s),s}H_{k,T(k,s),s}t_{k,T(k,s),s}X_{k,s}+\\ \sum_{k\prime \neq k,s\in U_{k\prime }}^{K}\left(\sqrt{\frac{p_{k\prime ,T(k\prime ,s),s}}{TL_{k,T(k\prime ,s)}}}\right)r_{k,T(k,s),s}H_{k,T(k\prime ,s),s}t_{k\prime ,T(k\prime ,s),s}X_{k\prime ,s}+\\ r_{k,s}n_{k,s} \end{array}$$

The first term in (2) is the desired MS signal, while the second term denotes Intra-CCI and Inter-CCI. Moreover, matrix **H***_k_*_,T(***k’***,*s*),*s*_ denotes the channel matrix of the *k*th MS with respect to the AP of the *k*’th MS and *TL* is the corresponding total losses (shadowing effects have been included as well). Each entry of the H*_k_*_,T(***k’***,*s*),*s*_ matrix has been calculated according to the latest 3GPP specifications for channel modeling [17]. Finally, r*_k_*_,T(*k*,*s*),*s*_ is the 1 × *M_r_* maximal ratio combining (MRC) multiplying vector and n*_k_*_,*s*_ is the *M_r_* × 1 noise vector.

## 3. Developed Massive MIMO Antenna Configuration

**Figure 2:**
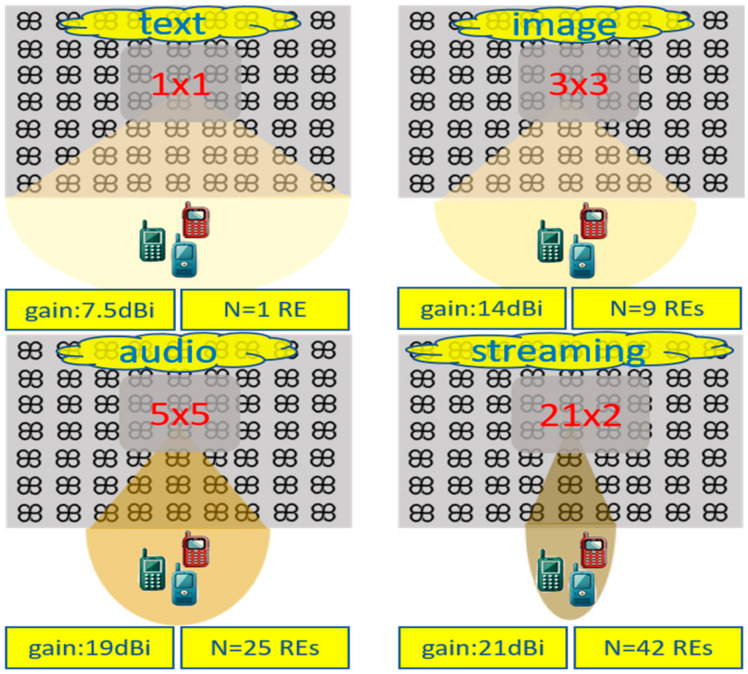
Illustration of the activation of the appropriate subarrays (1 × 1: single element, 3 × 3, 5 × 5, 21 × 2) according to three key requirements: spatial coverage, QoS and number of REs (*N*).

A 21 × 21 array of rounded crossed bowtie radiating elements (REs) is proposed, as illustrated in Figure 2. For the sake of space, Figure 2 shows an indicative antenna array consisting of 7 × 9 REs, while the actual antenna array architecture and its electromagnetic characteristics are thoroughly detailed in [18]. The REs used are notable for their low fabrication costs and straightforward manufacturing processes. The entire antenna array is designed to achieve a unidirectional radiation pattern and prevent energy loss through the back lobe. To this end, two ground planes are positioned beneath each RE at distances of λ_o_/4 and λ_o_/2, respectively (λ_ο_ is the carrier wavelength). The proposed REs mainly act as exciters to the reflector. Additionally, the aforementioned REs are rotated at ±45° to form a dual-polarized radiation pattern, which is commonly used in wireless communications, especially in cellular networks [19].

The rotation of these exciters, coupled with a 90° phase difference, promotes circular polarization, resulting in high cross-polarization power ratio (XPR) values exceeding 20 dB. These high XPR values ensure remarkable signal integrity, which is further enhanced by circular polarization as it eliminates the undesired high sensitivity of the receiver antenna orientation [20].

It is worth noting that the proposed antenna array configuration can support different phase activations for each RE. This can occur either across the entire array or within a subarray, supporting efficient beamforming techniques. To this end, the radiation pattern can be altered in both azimuth and elevation levels depending on the phase activation. This flexibility in beamforming allows for changes in the desired directions and gain [20]. In particular, the main radiation lobe can be steered around +30° and −30°, thereby enhancing the spatial coverage. In the same context, the activation of specific subarrays can lead to the formation of more directional radiation patterns, as shown in Figure 2, facilitating the support of different types of services. In this context, most of the related scenarios are differentiated according to the required energy consumption of the antenna array, which can be translated into the desired number of activated REs, the required quality of services (QoS), and finally, the required demand of spatial coverage.

Based on the previous categories, an efficient algorithm has been implemented to activate the appropriate radiating subarray. The algorithm is oriented to deliver the necessary services efficiently while minimizing energy usage. This is achieved by changing the number of REs according to the real-time demand of QoS without changing the required spatial coverage, if possible, as shown in Figure 2. This approach not only meets service requirements but also optimizes energy efficiency, thus improving overall network performance.

## 4. NOMA Transmission and Cooperative Beamforming in Cell-Free Massive MIMO Orientations

The parameters of the proposed resource allocation approach are summarized in Table 2, while the main approach is described in Algorithm 1. At the initial stage (Step 1), all VCs are initialized with the same number of PRBs (the available PRBs of the *b*th VC are stored in the set $S_{b}$, while the ones that are engaged for NOMA transmission are stored in the set $S_{NOMA}$). In the same context, all REs of each VC are initialized as well (the REs of the *l*th AP of the *b*th VC are stored in the set $RE_{b,l}$). In Step 2, MSs enter the network sequentially (it is assumed that they request a specific type of service (*R_k_* Mbps) that is translated to an equivalent number of *P* PRBs with a specific modulation order (MO) per PRB (*define_PRBs* function). For each MS that tries to access the network in the *b*th VC, the available PRBs are sorted according to their signal strength (Step 3), defined as the ratio of the equivalent squared channel matrix Frobenius norm to the corresponding total losses. These PRBs are derived from set $\left({S_{V}}_{b}\cup \bar{{S_{V}}_{b}}\right)\cap \bar{S_{b,NOMA}}$, where $V_{b}$ indicates all adjacent VCs with respect to the *b*th VC. In this case, it is apparent that the first term in the final set (i.e., ${S_{V}}_{b}\cup \bar{{S_{V}}_{b}}$) indicates that all PRBs are considered as available, even the occupied ones, as the algorithm tries to identify the optimum PRB per MS ($\bar{A}$ denotes the complementary set of A). To this end, the PRBs stored in the complementary set of $S_{b,NOMA}$ are considered as well. The sorted PRBs are stored in the set $U_{k,opt}$. If, however, there are no PRBs for downlink transmission, reject flat (*rf*) is set to 1 and the Monte Carlo (MC) simulation terminates.

In Step 4, for each PRB, power calculations take place. The algorithm initiates PRB assignment from the PRBs of the optimum set. However, if a PRB is already assigned to another MS, this is denoted as *k*’ (which means that $s_{opt}$ does not belong to the set of available PRBs $S_{b}$), and then PRB switching may need to take place. During PRB switching, the *k*th MS is allocated with the PRB that maximizes its signal strength (i.e., $s_{1}$ in Step 4 which equals $s_{opt}$), while the *k’*th MS that initially shares the same PRB with the *k*th MS is allocated with the next available one from its pool of PRB $U_{k\prime }$ (denoted as $s_{2}$). Hence, this new case (PRB switching, State 2) is compared to the case where the *k*th MS is allocated with a PRB from the available ones (i.e., stored in set $S_{b}$) and the *k*’th MS remains with the optimum PRB (i.e., $s_{opt}$), State 1. In both cases, the total downlink transmission power is evaluated. If PRB switching leads to reduced transmission power compared to State 1, then the corresponding sets are updated (i.e., the sets of allocated PRBs per MS) and the algorithm proceeds to Step 5 for power calculations. In the opposite case, NOMA transmission is examined. To this end, power calculations are repeated, where now both MSs share the same PRB (i.e., the set $U_{k}$ is updated with $s_{opt}$). Hence, a new NG is formulated, which includes the *k*th and *k’*th MSs (the corresponding index *ng* is updated as well, along with the corresponding NOMA set for the *b*th VC—$S_{b,NOMA}$).

To this end, two NOMA approaches are considered: In the first one, which will be denoted as soft NOMA (S-NOMA) throughout the rest of this work, NOMA transmission takes place only in the case where total downlink transmission power is reduced compared to the OMA case (i.e., State 2). In the second case, which will be denoted as hard NOMA (H-NOMA), the power comparison among NOMA/OMA cases is neglected. Hence, H-NOMA is adopted if it does not lead to power outage of at least one of the participating MSs. It becomes apparent that the fundamental difference between S-NOMA and H-NOMA lies in their inherent complexity, as in S-NOMA an additional power calculation step should be performed. It should be noted at this point that for every PRB assignment that takes place, the transmission vector matrix is calculated as the eigenvector that corresponds to the maximum eigenvalue of matrix **H**^H^**H** (i.e., *λ_m_*(**H**^H^**H**)).

In Step 5, the final power calculations take place for the considered beamforming configurations per AP and assigned PRBs. The goal is to select the optimum configuration per AP of the adjacent VCs to ensure QoS for all active MSs. If no power outage takes place, then on the one hand *rf* is set to 0 and on the other hand all corresponding PRB sets are updated in Step 6 (i.e., the set $MS_{b}$ includes all MSs of the *b*th VC, while the assigned PRBs to the *k*th MS are subtracted from $S_{b}$). Otherwise, additional beamforming configurations are examined. To this end, all available beamforming configurations of the *l*th AP of the *b*th VC are stored in the set $BC_{b,l}$. Hence, for every VC that belongs to the set of adjacent APs of the *b*th VC, a new beamforming configuration is iteratively selected from $BC_{b,l}$ and, in particular, the one with the minimum number of hardware elements. It should be noted at this point that beamforming configurations in each $BC_{b,l}$ set have been stored in ascending order. For every new potential beamforming configuration, power calculations are repeated until power outage does not take place. However, if acceptable QoS cannot be ensured, then *rf* is set to 1 and the new potential MS is rejected.

The MC simulation comes to an end either if power outage takes place in at least one VC (the corresponding threshold is set to *P_m_*) or if there are no available PRBs for downlink transmission, as previously mentioned (Step 3). In both cases, *rf* is set to 1. Otherwise, all corresponding sets are updated, and a new MS tries to enter the network in Step 2. For accurate performance evaluation, a large number of MC iterations is required. In this work, each scenario has been simulated for 10^4^ different snapshots with the help of a developed simulator that can execute MC simulations in parallel, as also described in the next section.
**Algorithm 1.** Resource allocation in cell-free massive MIMO NOMA orientations
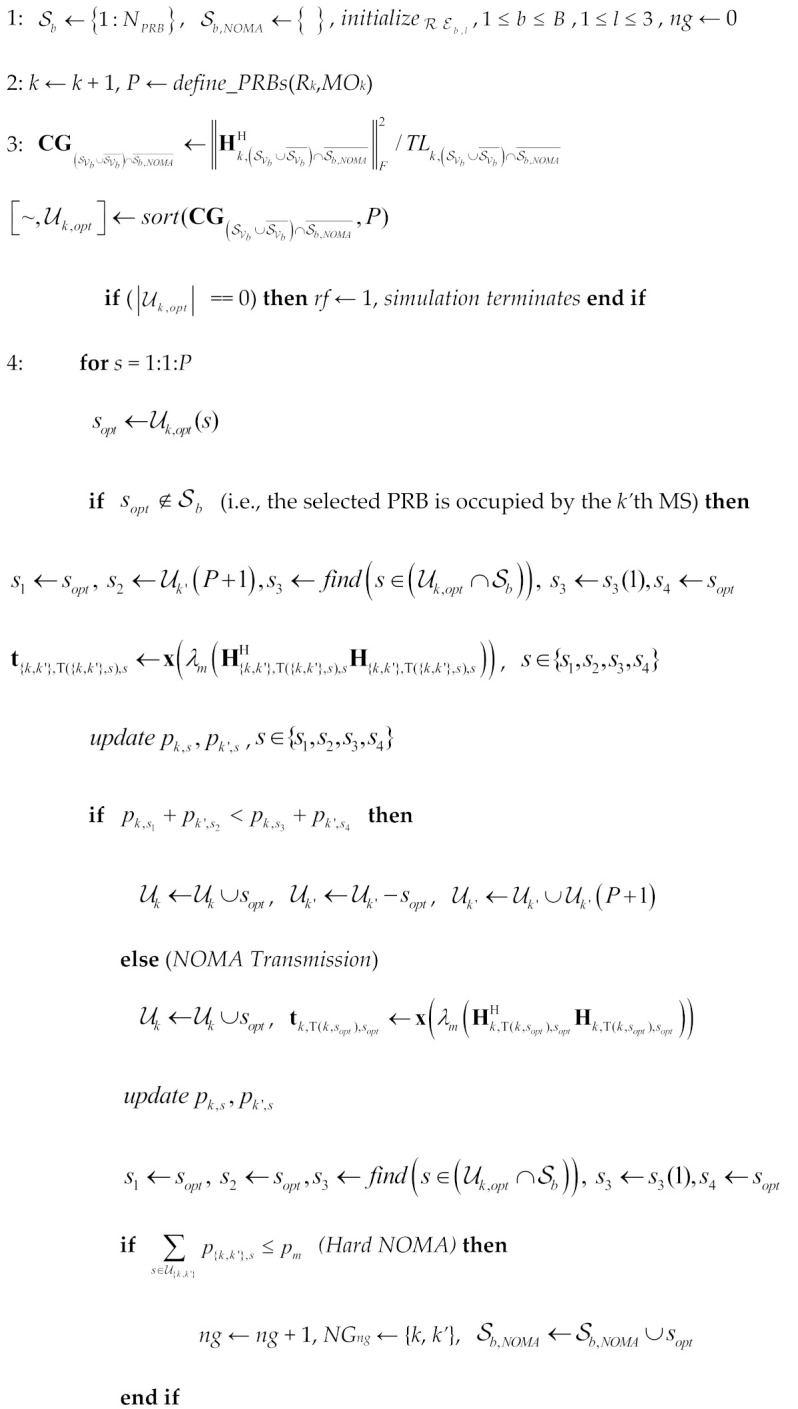

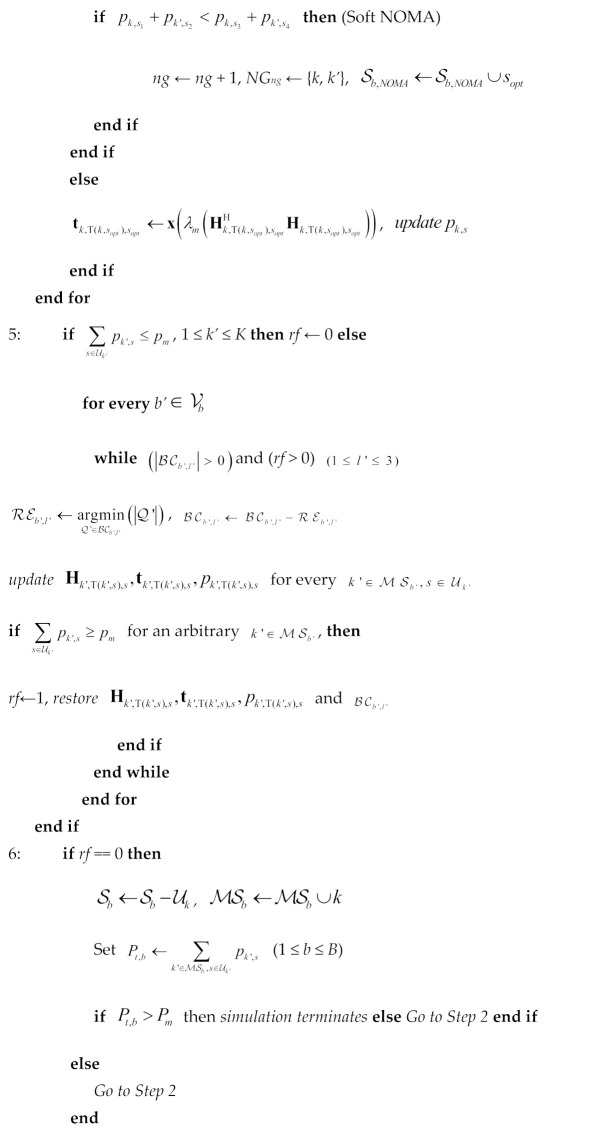


## 5. Results

**Figure 3:**
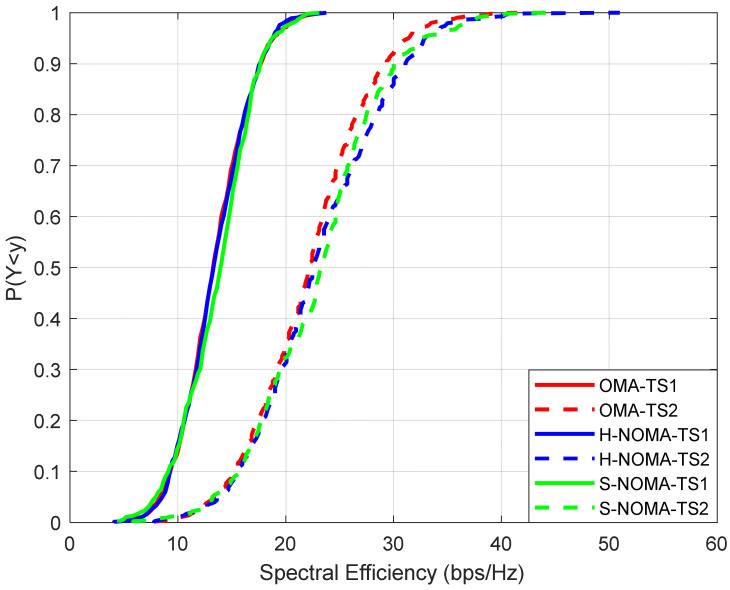
Cumulative distribution function of SE.

**Figure 4:**
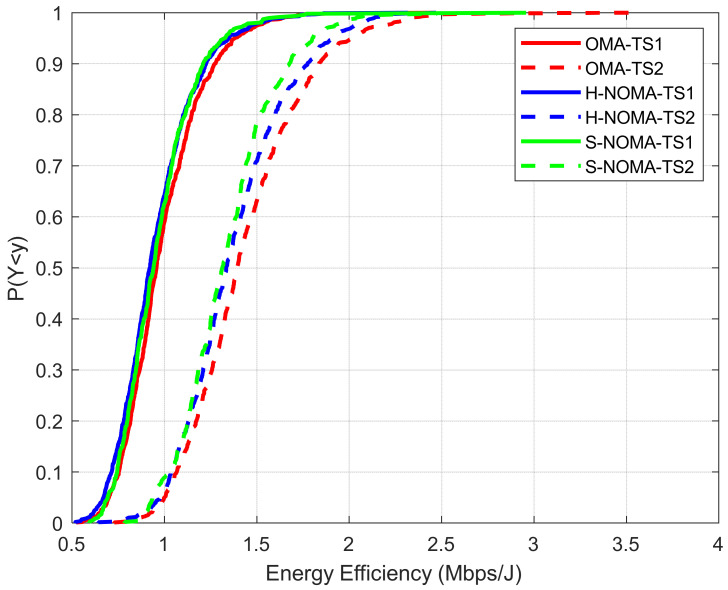
Cumulative distribution function of EE.

**Figure 5:**
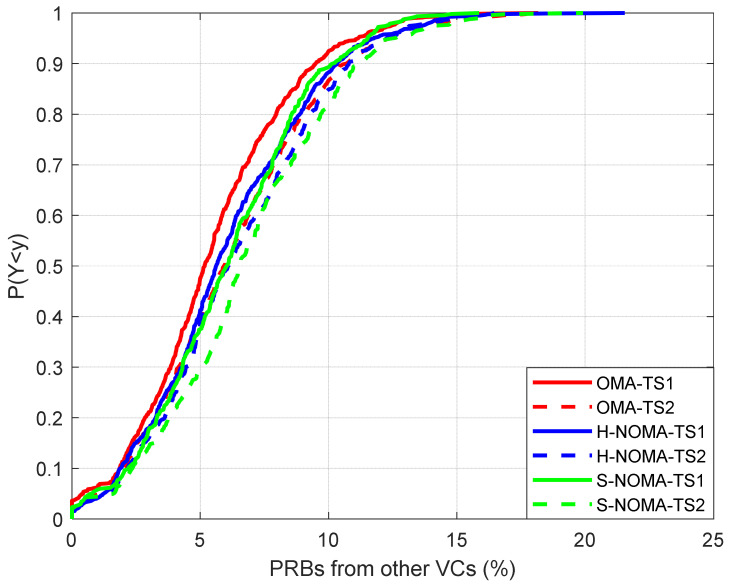
Cumulative distribution function of the available PRBs for downlink transmission from adjacent VCs.

**Figure 6:**
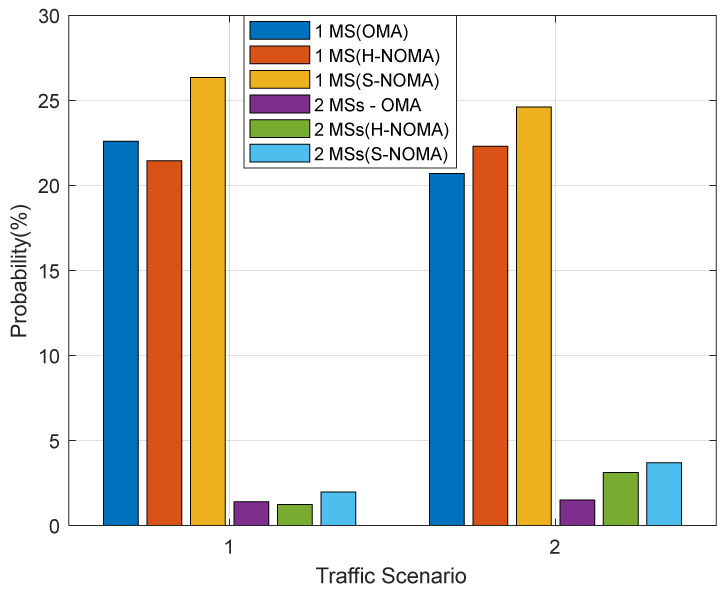
Probability of appearance of the minimum number of MSs.

**Figure 7:**
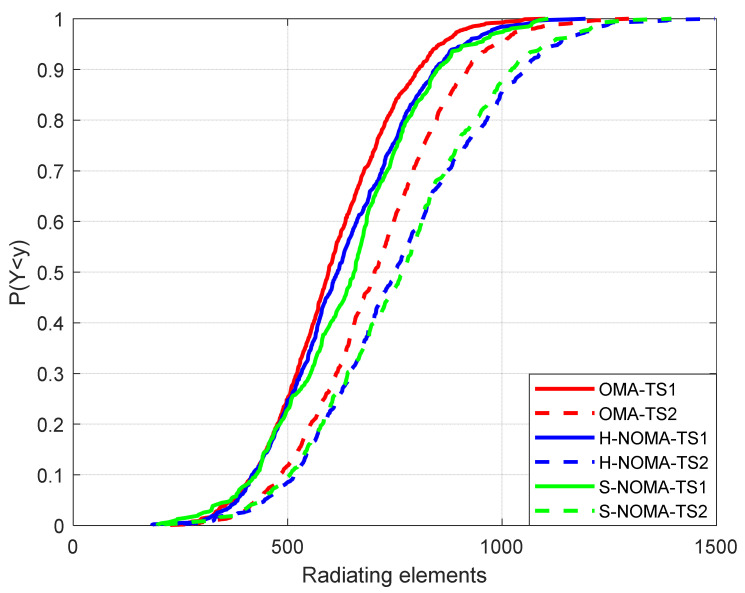
Cumulative distribution function of the number of REs.

The results are presented in Figure 3, Figure 4, Figure 5, Figure 6 and Figure 7. The output KPIs are SE, EE, the assigned PRBs from adjacent VCs with respect to the serving VC, the minimum number of MSs, and the number of REs in the topology. Moreover, two traffic scenarios (TSs) are considered: In the first case, QPSK modulation has been assumed per assigned PRB. In the second case, it is assumed that 50%/30%/20% of the MSs are assigned with QPSK/16-QAM/64-QAM modulations per PRB, respectively. As also described in Table 3 (simulation parameters), each MS is assumed to request 15 PRBs. Hence, corresponding transmission rates may vary from 21.6 Mbps (QPSK) to 64.8 Mbps (64-QAM). Throughout the rest of the paper, all output metrics will be compared with respect to their mean values, unless otherwise stated.

MSs enter the network sequentially, as also mentioned in the previous section. Performance is evaluated statistically, with the help of a developed system-level simulator that can execute MC simulations in parallel [9,18]. For each MC snapshot, and for each new MS that is conditionally admitted in the network, system-level parameters are initially calculated, such as channel matrices, total losses, etc. Afterwards, PRB assignment takes place, based on the proposed approach described in Algorithm 1. The potential MS is rejected if link outage takes place for another active MS. The MC simulation ends either if power outage occurs in one of the active APs or if there are no available PRBs for downlink transmission.

All the figures depict cumulative distribution function (CDF) curves. The performance of the proposed NOMA approach is compared with respect to the OMA case in [18] (CF m-MIMO orientation). As can be observed from Figure 3, SE can reach up to 13.5/22.2 bps/Hz for TSs 1 and 2, respectively (OMA). The increase in TS2 is expected, given the higher supported rates per MS. In H-NOMA transmission, the corresponding values are 13.5/22.6 bps/Hz, respectively. However, in S-NOMA, a further improvement of 4% in SE is achieved (TS2), as it reaches 23.2 bps/Hz. In this case, since NOMA is preferred only in the case where downlink transmission power is improved compared to the OMA case, more active MSs can be supported. It is important to note at this point that the results are improved with respect to [10], where SE does not exceed 15 bps/Hz, while they are also aligned with the ones in [22], for the same number of transmitting antennas per VC.

In Figure 4, the EE is presented. In general, the results are in agreement with the ones presented in [23]. As observed, EE does not decrease in the NOMA case for TS1, while a slight deterioration of approximately 4% is noted in TS2 (1.43/1.37 Mbits/J for the OMA/NOMA cases, respectively). This reduction is rather expected, due to the more directional beamforming configurations that are required for the support of the NOMA framework. Figure 5 depicts the percentage of PRBs that are assigned to MSs of other VCs with respect to the VC of interest. It becomes apparent that in the NOMA case, more PRBs are available for downlink transmission for both TSs, when compared to the OMA case. Specifically, this percentage reaches 5.5%/6.1/6.1% in TS1 for the OMA/H-NOMA/S-NOMA case, respectively, leading to an improvement of almost 11%. In TS2, the corresponding values are 6.2%/6.4%/6.8%.

Figure 6 depicts the minimum number of supported MSs in the topology, as this is measured from the corresponding probability of appearance. Two cases are considered, and in particular 1/2 MSs, respectively. As can be observed, this probability is significantly improved in TS2 for both cases of minimum MSs in NOMA transmission. In this operational mode, there are more PRBs available for downlink transmission compared to the OMA case, as previously mentioned, which in turn leverage spatial coverage. Notably, this probability improves in the S-NOMA case, due to the more effective downlink transmission power management. Specifically, for one MS (TS2), this probability reaches 1.4%, 1.2%, and 2.1% for the OMA/H-NOMA/S-NOMA cases, respectively. It becomes apparent that this probability is significantly increased in the S-NOMA case, when compared to OMA. For two MSs, the corresponding values are 1.5%, 3.1%, and 3.7%, leading to a further probability improvement compared to the previous case. Similar conclusions can be drawn in the case of TS1 as well. For one MS, this probability reaches 22.6%, 21.4%, and 26.4% for the OMA/H-NOMA/S-NOMA cases, respectively. For two MSs, the corresponding values are now 20.7%/22.2%/24.6%. Hence, the improvement in probability when comparing the S-NOMA/OMA cases is almost 19%.

Finally, Figure 7 illustrates the total number of REs in the topology. As expected, this number increases in NOMA transmission for high data rate services, since supporting a greater number of PRBs with higher modulation orders inevitably leads to more directive radiation patterns. However, the increase in hardware complexity, which is directly related to the total number of active REs, is offset by the corresponding increase in the number of available PRBs for downlink transmission and the minimum number of MSs in the topology. Specifically, the increase is limited to 5% for TS1 and NOMA transmission (626/636/605 REs for the H-NOMA/S-NOMA/OMA cases, respectively) and 7.5% for TS2 (758/758/705 REs for the H-NOMA/S-NOMA/OMA cases, respectively).

## 6. Conclusions

The performance of a cooperative adaptive beamforming approach was evaluated in combination with NOMA transmission and cell-free resource allocation. In this context, large-scale mmWave broadband networks were considered. Rather than selecting the optimal pair of NOMA users based on channel characteristics and distance, each user is assigned the physical resource block that maximizes its signal strength. Cooperative beamforming is then employed to mitigate the effects of co-channel interference. As a result, various performance metrics were improved, including the minimum number of active users in the topology and available physical resource blocks for downlink transmission, all with minimal hardware overhead.

Overall, the presented results can be quite promising when working towards effective resource management in highly dense and complex network orientations. On the one hand, the multitude of available radiation patterns in cell-free massive MIMO antenna orientations facilitates efficient resource optimization in multiuser scenarios, which is further improved with the help of NOMA. To this end, minimum transceiver complexity is required since beamforming configurations are a priori defined. On the other hand, spectrum efficiency is also improved with minimum computational burden, since instead of trying to identify the optimum NOMA pair of mobile users per case, which can be an NP-hard problem, a NOMA group can be directly formulated consisting of two users whose channel gain is maximized for a particular resource block.

Future work will focus on deploying machine learning techniques for selecting the optimal beamforming configuration per AP, as well as exploring cooperative NOMA strategies. Finally, a challenging issue is the deployment of intelligent reflecting surfaces that can leverage near-edge connectivity.

## Figures and Tables

**Table 1:** Categorization of indicative presented studies.

Related Works	Key ΝOΜA Considerations	Limitations—Open Issues
[7]	Similarity between user channels based on the Euclidean distance	Single-cell scenario Per user optimum channel assignment is not considered
[8]	NOMA grouping for MEC server and local helper	Single cell scenario
[9]	NOMA grouping based on the maximization of signal to interference and signal to jamming ratio	Massive MIMO systems and adaptive beamforming techniques have not been considered SINR and SJR calculations can be time consuming
[10,11]	NOMA in IRS cell-free m-MIMO systems Machine learning-assisted user pairing	Single-cell performance evaluation
[12]	NOMA with spatial multiplexing	Single-cell performance evaluation
[13]	Tier-based NOMA clustering	Single-cell performance evaluation Mixed types of services per user are not supported
[14]	DRL-based NOMA	Increased computational complexity
[15]	NOMA in relay-aided networks	Massive MIMO configurations for interference management

**Table 2:** Algorithm’s parameters.

Parameter	Variable
Available PRBs in the *b*th VC	$S_{b}$
PRBs in NOMA transmission in the *b*th VC	$S_{b,NOMA}$
REs of the *l*th AP of the *b*th VC	$RE_{b,l}$
Adjacent VCs of the *b*th VC	$V_{b}$
Available PRBs of the adjacent APs of the *bt*h VC	$S_{V_{b}}$
Beamforming configurations of the *l*th AP of the *b*th VC	$BC_{b,l}$
The MSs in the *b*th VC	$MS_{b}$
Channel gain matrix	**CG**
Maximum eigenvalue of matrix **A**	*λ_m_*(**A**)
Eigenvector of matrix **A** for the *λ*^th^ eigenvalue	**x**(*λ*)

**Table 3:** Simulation parameters.

Parameter	Value or Assumption
Number of virtual cells (*B*)	19
Access points per virtual cell	3
Cell radius (*m*)	500
Total bandwidth (MHz)	100
Pathloss model [17]	UMa
Carrier frequency (GHz)	28
Subcarrier spacing (kHz)	60
Subcarriers per PRB	12
PRBs per VC	132
Number of clusters/Subpaths per cluster [17]	6/20
Requested PRBs per MS	15
Monte Carlo snapshots per scenario	10^4^
Antenna elements per MS (*M_r_*)	2
Maximum transmission power per BS/MS (*P_m_*/*p_m_*) (W)	20/1
Required E_b_/N_o_ (dB) for QPSK/16-QAM/64-QAM modulation	9.6/16.4/22.7 [21]
Transmission rate per MS for QPSK/16-QAM/64-QAM modulation (Mbps)	21.6/43.2/64.8

## Data Availability

Data are contained within the article.

## Abbreviations

The following abbreviations are used in this manuscript:

5G	fifth generation
3GPP	Third Generation Partnership Project
6G	sixth generation
AP	access point
CCI	co-channel interference
CDF	cumulative distribution function
CF	cell free
DL	deep learning
DRL	deep reinforcement learning
EE	energy efficiency
eMBB	enhanced mobile broadband
Inter-CCI	intercell co-channel interference
Intra-CCI	intracell co-channel interference
IoT	Internet of Things
IRS	intelligent reflecting surface
KPI	key performance indicator
MEC	multi-access edge computing
MC	Monte Carlo
MO	modulation order
mMTC	massive machine-type communications
m-MIMO	massive multiple input multiple output
mmWave	millimeter wave
MRC	maximal ratio combining
MS	mobile station
NFV	network function virtualization
NOMA	non-orthogonal multiple access
NG	NOMA group
OMA	orthogonal multiple access
PRB	physical resource block
QoS	quality of service
RA	resource allocation
RE	radiating element
SE	spectral efficiency
SJR	signal to jamming ratio
SIC	successive interference cancelation
SINR	signal to interference plus noise ratio
TL	total losses
TS	traffic scenario
UAV	unmanned aerial vehicle
UDN	ultra dense networks
URLLC	ultra-reliable low latency communications
VC	virtual cell
XPR	cross-polarization power ratio

## References

[1] Liu Y., Clerckx B., Popovski P. (2024). Network slicing for eMBB, URLLC, and mMTC: An uplink rate-splitting multiple access approach. IEEE Trans. Wireless Commun..

[2] Albreem M.A., Juntti M., Shahabuddin S. (2019). Massive MIMO detection techniques: A Survey. IEEE Commun. Surv. Tutor..

[3] Wang X. (2018). Millimeter wave communication: A comprehensive survey. IEEE Commun. Surv. Tutor..

[4] Makki B., Chitti K., Behravan A., Alouini M.-S. (2020). A survey of NOMA: Current status and open research challenges. IEEE Open J. Commun. Soc..

[5] Lu Y., Zhang P., Duan Y., Guizani M., Wang J., Li S. (2024). Dynamic scheduling of IoV edge cloud service functions under NFV: A multi-agent reinforcement learning approach. IEEE Trans. Veh. Tech..

[6] Tinh B.T., Nguyen L.D., Kha H.H., Duong T.Q. (2022). Practical optimization and game theory for 6G ultra-dense networks: Overview and research challenges. IEEE Access.

[7] Jawarneh A., Kadoch M., Albataineh Z. (2022). Decoupling energy efficient approach for hybrid precoding-based mmWave massive MIMO-NOMA with SWIPT. IEEE Access.

[8] Khazali A., Bozorgchenani A., Tarchi D., Shayesteh M.G., Kalbkhani H. (2023). Joint task assignment, power allocation and node grouping for cooperative computing in NOMA-mmWave mobile edge computing. IEEE Access.

[9] Gkonis P., Trakadas P., Sarakis L., Giannopoulos A., Spantideas S., Capsalis N. On the performance evaluation of 5G MIMO networks employing NOMA via system-link level simulations. Proceedings of the IEEE 9th International Conference on Information, Communication and Networks (ICICN).

[10] Dang X.-T., Nguyen H.V., Shin O.-S. (2023). Optimization of IRS-NOMA-assisted cell-free massive MIMO systems using deep reinforcement learning. IEEE Access.

[11] Perdana R.H.Y., Nguyen T.-V., An B. (2023). Adaptive user pairing in multi-IRS-aided massive MIMO-NOMA networks: Spectral efficiency maximization and deep learning design. IEEE Trans. Commun..

[12] Wang J., Liu Y., Mu X., Ma X., Liu W., Xie W. (2024). Joint subchannel and power allocation in NOMA-based spatial modulation systems. IEEE Trans. Wirel. Commun..

[13] Tai T.V., Xuan Uyen N.T., Khoa D.L. Optimal user clustering and power allocation in NOMA systems. Proceedings of the International Conference on Advanced Technologies for Communications (ATC).

[14] Lim B., Yun W.J., Kim J., Ko Y.-C. (2024). Joint user clustering, beamforming, and power allocation for mmWave-NOMA with imperfect SIC. IEEE Trans. Wirel. Commun..

[15] Özduran V., Nomikos N., Soleimani-Nasab E., Ansari I.S., Trakadas P. (2024). Relay-aided uplink NOMA under non-orthogonal CCI and imperfect SIC in 6G Networks. IEEE Open J. Veh. Technol..

[16] Amhaz A., Elhattab M., Sharafeddine S., Assi C. (2024). UAV-assisted cooperative downlink NOMA: Deployment and resource allocation. IEEE Trans. Veh. Tech..

[17] (2019). Study Channel Model for Frequencies From 0.5 to 100 GHz, Version 14.3.0, Release 14.

[18] Gkonis P., Lavdas S., Vardoulias G., Trakadas P., Sarakis L., Papadopoulos K. (2024). System level performance assessment of large-scale cell-free massive MIMO orientations with cooperative beamforming. IEEE Access.

[19] Zheng W.C., Zhang L., Li Q.X., Leng Y. (2014). Dual-band dual-polarized compact bowtie antenna array for anti-interference MIMO WLAN. IEEE Trans. Antennas Propag..

[20] Balanis C. (2016). Antenna Theory: Analysis and Design.

[21] Giuliano R., Monti C., Loreti P. (2008). WiMAX fractional frequency reuse for rural environments. IEEE Wireless Commun..

[22] El-ghorab M.A., El-meligy M.R., Ibrahim M.M., Newagy F. (2022). Energy-efficient user pairing for downlink NOMA in massive MIMO networks. Appl. Sci..

[23] Aldebes R., Dimyati K., Hanafi E. (2022). Genetic algorithm for optimizing energy efficiency in downlink mmWave NOMA system with imperfect CSI. Symmetry.

